# Measuring mistreatment of women during childbirth: a review of terminology and methodological approaches

**DOI:** 10.1186/s12978-017-0403-5

**Published:** 2017-10-26

**Authors:** Virginia Savage, Arachu Castro

**Affiliations:** Department of Global Community Health and Behavioral Sciences, Tulane University School of Public Health and Tropical Medicine, 1440 Canal Street, Mail Code #8319, New Orleans, LA 70112 USA

**Keywords:** Mistreatment, Violence, Obstetric violence, Gender-based violence, Disrespect and abuse, Dehumanization of care, Maternal health care, Medicalization, Measurement, Latin America and the Caribbean

## Abstract

**Background:**

Although mistreatment of women during facility-based childbirth has received increasing recognition as a critical issue throughout the world, there remains a lack of consensus on operational definitions of mistreatment and best practices to assess the issue. Moreover, only minimal research has focused on mistreatment in Latin America and the Caribbean, a region notable for social inequalities and inequitable access to maternal health care.

**Methods:**

In this article, we discuss the results of a literature review that sought to contribute to the determination of best practices in defining and measuring the mistreatment of women during childbirth, particularly within Latin America and the Caribbean. The review includes a total of 57 English, Spanish, and Portuguese-language research publications and eight legal documents that were published between 2000 and 2017.

**Results:**

While the typologies of “disrespect and abuse” and “mistreatment during facility-based childbirth” are most frequently employed in global studies, “obstetric violence” remains the most commonly operationalized term in Latin America and the Caribbean in both research and policy contexts. Various researchers have advocated for the use of those three different typologies, yet the terms all share commonalities in highlighting the medicalization of natural processes of childbirth, roots in gender inequalities, parallels with violence against women, the potential for harm, and the threat to women’s rights. For measuring mistreatment, half of the research publications in this review use qualitative methods, such as in-depth interviews and focus groups. After analyzing the strengths and limitations of quantitative, qualitative, and mixed methods approaches to assessing mistreatment, we recommend mixed methods designs as the optimal strategy to evaluate mistreatment and advocate for the inclusion of direct observations that may help bridge the gap between observed measures and participants’ self-reported experiences of mistreatment.

**Conclusions:**

No matter the conceptual framework used in future investigations, we recommend that studies seek to accomplish three objectives: (1) to measure the perceived and observed frequencies of mistreatment in maternal health settings, (2) to examine the macro and micro level factors that drive mistreatment, and (3) to assess the impact of mistreatment on the health outcomes of women and their newborns.

## Abstract in Spanish

**Antecedentes:**

Aunque el maltrato durante la atención del parto en establecimientos de salud ha recibido atención como un problema crítico en todo el mundo, no existe un consenso ni sobre las definiciones operativas del maltrato ni sobre las mejores prácticas para evaluar el problema. Sólo algunas investigaciones se centran en el maltrato en América Latina y el Caribe, una región que se caracteriza por las desigualdades sociales y el acceso desigual a la atención materna en salud.

**Métodos:**

Presentamos los resultados de una revisión bibliográfica que contribuye a determinar las mejores prácticas en la definición y la medición del maltrato de las mujeres durante el parto. Generamos información que puede informar la selección de mejores prácticas en la medición del maltrato, en particular en América Latina y el Caribe. La revisión incluye un total de 57 publicaciones y ocho documentos legales en inglés, español y portugués publicados entre 2000 y 2017.

**Resultados:**

Aunque muchas investigaciones usan las tipologías de “falta de respeto y abuso” y "maltrato durante la atención del parto,” el concepto de “violencia obstétrica” es el término más común en contextos de investigación y de política en América Latina y el Caribe. A pesar de sus diferencias, los tres conceptos comparten elementos en sus definiciones, como la medicalización de los procesos naturales del parto, sus raíces en las desigualdades de género, los paralelos con la violencia contra la mujer, el potencial de daño y la amenaza a los derechos de las mujeres. Para medir el maltrato, la mitad de las publicaciones en esta revisión utilizan métodos cualitativos, como entrevistas en profundidad y grupos focales. Después de analizar las ventajas y limitaciones de los métodos cuantitativos, cualitativos y mixtos, recomendamos el uso de diseños de métodos mixtos para evaluar el maltrato y la inclusión de observaciones directas para superar la brecha entre las medidas observadas y las experiencias reportadas de maltrato.

**Conclusiones:**

Con independencia del método o del marco conceptual, recomendamos que las investigaciones futuras alcancen tres objetivos: (1) medir la frecuencia reportada y la observada del maltrato en establecimientos de salud materna, (2) examinar los factores macro y micro que causan el maltrato y (3) evaluar el impacto del maltrato en los resultados en salud de las mujeres y sus recién nacidos.

## Plain English summary

Although mistreatment of women giving birth in medical facilities has received increasing global attention, researchers have not yet agreed on a singular definition of mistreatment or best practices in measuring it. By examining the contexts, strengths, and limitations of different investigations, this review generates information to contribute to the determination of best practices in defining and measuring mistreatment, particularly within Latin America and the Caribbean, where minimal research has been conducted. Many definitions of mistreatment used in existing research draw from three concepts: “disrespect and abuse,” “mistreatment of women during facility-based childbirth,” and “obstetric violence.” Although these concepts have distinct definitions and systems to classify the different forms of mistreatment, all three concepts highlight the connection between mistreatment and other forms of gender violence, the medicalization of natural processes of childbirth, roots in gender inequalities, and the threat to women’s rights and health. Considering the results of different investigations that sought to measure mistreatment, we found that mixed method approaches are able to gain the most comprehensive information and we recommend that future studies incorporate direct observations to account for gaps that have been documented between perceived and observed frequency of mistreatment. Overall, we recommend that future studies seek to: (1) measure the perceived and observed frequencies of mistreatment in maternal health settings, (2) examine the macro and micro level factors that drive mistreatment, and (3) assess the impact of mistreatment on the health outcomes of women and their newborns.

## Background

Numerous research studies have begun to document mistreatment during facility-based childbirth as an urgent issue that affects women throughout the world [1]. However, despite the increased recognition of the issue, mistreatment of women during childbirth, also labeled as obstetric violence, dehumanized care, or disrespect and abuse, we argue that it continues to be a nascent area of study and several gaps remain in existing literature. First, there has been minimal discussion regarding best practices in measuring mistreatment. Currently, there are several publications that have proposed definitions and conceptual frameworks for understanding mistreatment [2–5]. The World Health Organization (WHO) also released a statement in 2015 that emphasized that “every woman has the right to the highest attainable standard of health, which includes the right to dignified, respectful health care” [6], and identified five areas of action in which researchers, policymakers, and health professionals should work to reduce mistreatment: (1) increasing support for research and action, (2) creating programs to promote respectful, high quality maternal health care, (3) developing rights-based frameworks for action, (4) generating data on the prevalence of disrespect and abuse and interventions to mitigate it, and (5) driving intersectional initiatives that encourage the participation of women [6]. The WHO has since developed tools with a new typology for classifying mistreatment based on results from an extensive, systematic literature review, which mark the first attempt to standardize the measurement of mistreatment in different clinical settings throughout the world [3, 7]. Although the WHO is conducting research in Ghana, Guinea, Nigeria, and Myanmar to inform the design of a direct observation tool and survey instrument that will be tested in the second phase [7], researchers have yet to reach a consensus on best practices or standardized tools to measure mistreatment.

Second, existing studies on mistreatment have been geographically limited. In particular, few studies have purposively examined mistreatment in Latin America and the Caribbean, a region notable for social inequalities and inequitable access to quality maternal health care [2, 8]. While many countries in Latin America and the Caribbean have enacted measures to promote universal health coverage, a recent UNICEF-Tulane University report documented vast inequities in maternal and reproductive health throughout the region that stem from social inequalities between the wealthy and the impoverished, between women of high and low levels of education, between dominant and minority ethnic groups, and between urban and rural locations of residence [8]. Social inequalities are also reflected in the organization of national health systems in various Latin American and Caribbean countries, where health systems are organized into well-funded and well-resourced social security systems for those formally employed and into subsidized insurance systems for those without formal employment that are overseen directly by Ministries of Health—that often operate their own health facilities with lower levels of funding and resources, as well as with lower quality services [9]. Many countries are working to reform these systems, yet the social segmentation of health services remains a barrier to the achievement of universal health coverage [9], and creates unique contexts in which the mistreatment of women during childbirth may take place.

This article aims to contribute to the determination of best practices in defining and measuring the mistreatment of women during childbirth. Drawing from an extensive review of research literature and legal documents, subsequent sections of the paper explore the contexts, strengths, and limitations of the working definitions of mistreatment as well as the various methodologies that have been used to measure mistreatment in clinical settings throughout the world. Although this review includes research from all world regions, a particular focus is given to implications for the study of mistreatment in Latin America and the Caribbean. By analyzing the strengths and limitations of different methodological approaches, this article seeks to provide practical insight for the development of future research and programmatic initiatives that seek to measure the frequency and magnitude of mistreatment of women during childbirth.

## Methods

In this article, we document the results of a literature review that explored the working definitions of mistreatment of women in childbirth as well as the methodologies with which mistreatment has been assessed in previous research studies throughout the world. We conducted searches on Pubmed, Google Scholar, and Scielo, and only retained literature published between the years 1998–2017 in order to narrow our focus to current working definitions and methodologies in the studies of mistreatment. Searches included combinations of the following key words: in English, *disrespect and abuse, childbirth, discrimination, humanization of childbirth, humanized care, institutional violence, maternal health care, mistreatment, obstetric violence, respectful care*; in Spanish, *abuso, atención en salud materna, discriminación, maltrato, parto humanizado, parto respetado, violencia, violencia obstétrica, salas de parto*; in Portuguese, *abuso, desrespeito, humanização do nascimento, maternidades, parto, saúde materna, violência institucional, violência obstétrica*.

Key word searches and snowball searches generated 57 articles—6 in Portuguese, 4 in Spanish, and 47 in English—That were eligible for synthesis and 8 documents with legal definitions of obstetric violence or related concepts that exist in Latin America and the Caribbean. Eligibility was limited to research studies that purposefully sought to: (a) define mistreatment, (b) measure the frequency of mistreatment, (c) determine the most prevalent forms of mistreatment in specific contexts, (d) examine the drivers of mistreatment, (e) assess the effects of mistreatment on women’s health outcomes or health behaviors, or (f) gather data on women and health care in Latin America and the Caribbean. In total, 65 articles and documents were included in the final analysis. The synthesis excluded full-text articles that discovered or documented mistreatment in the context of studying quality of maternal health care or access to care in general. The synthesis also excluded articles that examined mistreatment or discrimination within other health care settings (such as sexual and contraception health clinics, community health centers, or areas of hospitals not related to maternal health), and among other populations (such as men, children, or the elderly). Searches and analysis took place between January 2016 and May 2017.

## Terminology and Definitions of mistreatment

This section includes a total of 21 studies, statements, commentaries, and 7 legal documents published between 1998 and 2017 that seek to define some aspect of mistreatment in clinical maternal health care settings. The majority of these publications (19) were published in English—of which 8 were specific to Latin America—8 in Spanish, and 1 in Portuguese. The search for legal documents was limited to Latin America and included documents from Argentina, Brazil, Chile, Costa Rica, El Salvador, Mexico, and Venezuela.

Within the past two decades, the mistreatment of women during childbirth has been labeled and defined in various ways. Publications included in our review have referred to the phenomenon most frequently as “mistreatment of women in childbirth at health facilities,” “obstetric violence,” “disrespect and abuse,” “institutional violence,” and “dehumanized birth,” among other terms. While these labels have been used interchangeably at times, several authors have argued for the recognition of nuances distinct to each term, and increasing debate has arisen about what to call mistreatment and how to construct a concise, yet comprehensive definition that may be operationalized for the development of study tools and health service assessments.

In 2010, as part of the United States Agency for International Development (USAID)‘s Translating Research into Action project, researchers Bowser and Hill published a landscape analysis that synthesized existing research concerning “disrespect and abuse in facility-based childbirth” [5]. One of the first comprehensive reviews on the topic, the report proposed seven categories to organize the various forms of disrespect and abuse documented by previous studies: (1) physical abuse, (2) non-consented care, (3) non-confidential care, (4) non-dignified care (including verbal abuse), (5) discrimination based on specific patient attributes, (6) abandonment of care, and (7) detention in facilities [5]. Since the publication of that framework, those seven categories have constituted the conceptual basis for various studies that are discussed in subsequent sections of this article, and were referenced by the WHO in their 2015 *Statement on the prevention and elimination of disrespect and abuse during facility-based childbirth* [6].

Bowser and Hill’s seven categories of disrespect and abuse also formed the basis for another international declaration, the White Ribbon Alliance’s *Respectful Maternity Care Charter: the Universal Rights of Childbearing Women* in 2011 [10]. Created by a multi-stakeholder group of researchers and leaders from the WHO, USAID, Family Care International, International Confederation of Midwives, and other international organizations, the *Respectful Maternity Care Charter* draws from international mandates to create a list of seven rights that should be guaranteed to all women during pregnancy and childbirth, and that specifically address the seven categories of disrespect and abuse listed by Bowser and Hill [10]. For example, to prevent physical abuse against women during childbirth, the *Respectful Maternity Care Charter* declares that women have the right to “freedom from harm and ill treatment” [10]. Other rights listed in the charter include confidentiality and privacy, freedom from discrimination, information and informed consent, dignity and respect, timely care, and self-determination and autonomy [10]. These rights have also served as a framework for numerous study tools and interventions that seek to reduce disrespect and abuse during facility-based childbirth.

However, despite the widespread use of Bowser and Hill’s categories of disrespect and abuse, various researchers have highlighted important limitations to those definitions. In their 2014 review, Freedman et al. argued that the seven categories do not sufficiently differentiate between the forms of disrespect and abuse that stem from individual behaviors and the forms that arise from health system deficiencies [4]. These authors expanded on the seven categories, and created a new framework that connects individual, structural, and policy level drivers of disrespect and abuse with the perceptions and norms of health care providers and women using clinical maternity services [4]. For example, individual level factors include “behavior that all agree constitutes disrespect and abuse” and “normalized disrespect and abuse: behavior that women consider disrespect and abuse but providers do not. Behavior that women consider normal or acceptable but others consider disrespect and abuse” [4]. Structural level factors include: “poor treatment or conditions caused by system deficiencies and considered disrespect and abuse by women and providers” and those same issues which are considered to be acceptable [4]. Finally, policy level drivers include “deviations from national standards of good quality care” and “deviations from human rights standards” [4]. In 2015, WHO researchers Bohren and colleagues also highlighted limitations in Bowser and Hill’s model, citing that the seven categories of disrespect and abuse lack operational definitions that can be standardized and comparable between investigations [3]. Seeking to construct such operational definitions, they published a systematic review that synthesized 65 English, Spanish, French, and Portuguese-language publications and proposed a classification system for the “mistreatment of women in childbirth at health facilities” [3]. Their evidenced-based typology contains seven overarching categories of mistreatment, with several second and first order groups within the broader categories: (1) physical abuse**,** including use of force and physical restraint, (2) sexual abuse, including second and first order categories of the same name, (3) verbal abuse, including harsh language, threats, and blaming, (4) stigma and discrimination, including discrimination based on sociodemographic characteristics and medical conditions, (5) failure to meet professional standards of care, including lack of informed consent and confidentiality, physical examinations and procedures, and neglect and abandonment, (6) poor rapport between women and providers, including ineffective communication, lack of supportive care, and loss of autonomy, and (7) health system conditions and constraints, including lack of resources, lack of policies, and health facility culture [3]. In addition to these categories, the 2015 review emphasized that mistreatment may stem from both intentional and unintentional actions of medical providers, as well as from conditions within health systems and facilities [3].

Bohren et al. argue that “mistreatment” is a more inclusive term than “disrespect and abuse” given its broader scope of categories and emphasis on different sources of mistreatment [3]. WHO researchers have since been using the mistreatment typology as the basis for instruments in the study that examines mistreatment in Ghana, Guinea, Nigeria, and Myanmar, that is described elsewhere in this article [7]. Ultimately, the WHO researchers hope the mistreatment typology will enable the development of assessment tools that can standardize the measurement of mistreatment worldwide [11]. However, the mistreatment typology has not gone without criticism; in their 2015 essay, researchers Jewkes and Penn-Kekana argue that the definitions may in fact be too broad for operationalization and that the mistreatment typology would benefit from a more narrow focus on intentional use of violence and structural deficiencies that amount to violence [1].

### Terminology and definitions of mistreatment in Latin America and the Caribbean

Within Latin America and the Caribbean, the majority of legal and research discussions have not centered on mistreatment or disrespect and abuse, but rather on terminology related to obstetric violence, dehumanized care, and discrimination against certain populations in clinical settings. For example, a study by Castro et al., based on a review of 60 publications, identified six dimensions of mistreatment that specifically affect indigenous and Afrodescendant women in Latin America and the Caribbean during pregnancy and childbirth and that need to be understood and addressed as key drivers of inequitable health outcomes: “patient-blaming, purposeful neglect, verbal or physical abuse, disregard for traditional beliefs, and the non-use of indigenous languages for patient communication. These obstacles prevent delivery of appropriate and timely clinical care, and also produce fear of shame, abuse, or ineffective treatment, which, in addition to financial barriers, deter women from seeking care” and fuel inequitable health outcomes between minority and dominant ethnicities [2].

An emphasis on violence has been particularly common among definitions of mistreatment that have emerged from studies and policies in Latin America and the Caribbean. At least since the 1990s, regional research started to focus on mistreatment as a form of violence or abuse that resembled other forms of violence against women, with some forms specific to clinical maternity settings, such as unnecessary cesareans or episiotomies and unconsented intrapartum sterilizations [12–17]. Some of these studies also discussed institutional and structural violence from health systems that reflects gender inequalities and power hierarchies within health facilities [15, 17].

Subsequently, in 2007, Venezuela became the first nation in the world to legally define and outlaw “obstetric violence,” which was outlined in the country’s Organic Law on the Rights of Women to a Life Free of Violence as: “the appropriation of women’s body and reproductive processes by health personnel, which is expressed by a dehumanizing treatment, an abuse of medicalization and pathologization of natural processes, resulting in a loss of autonomy and ability to decide freely about their bodies and sexuality, negatively impacting their quality of life” [18]. The law also specified acts that would constitute obstetric violence including: obstructing the early attachment of the child with his/her mother, performing cesareans that were not medically indicated or consented, and restricting women’s choices of birth positions, among other acts [18]. Since the ratification of the law, Argentina has also passed similar legislation defining and outlawing obstetric violence [19], as have various states in Mexico [20]. Other countries such as Chile and Costa Rica have introduced relevant legislature, though they have not yet become law [21, 22]. Furthermore, Brazil and Argentina have passed legislation demanding the “humanization of childbirth” [23, 24] and El Salvador passed the Law of Gestational Security and the Empowerment of Child Development in 2014, which calls for dignified treatment, humanized care, cultural appropriateness, and freedom from discrimination in maternal and reproductive health services [25].

Most definitions of obstetric violence in Latin America and the Caribbean have emphasized the medicalization of the natural processes of childbirth and unbalanced power dynamics between health personnel and women in labor. In their 2016 review, Sadler and colleagues argued that “although it has been often used as a synonym for disrespect, abuse and mistreatment during childbirth […] obstetric violence has the potential for addressing the structural dimensions of violence within the multiple forms of disrespect and abuse” and—highlighting the grassroots origins of the term as well as its explicit connection with gender violence—they advocate for the use of obstetric violence as a central concept for future studies and interventions [26]. With this expanded emphasis on structural dimensions, the authors suggest that obstetric violence would be the best term to accurately convey mistreatment as forms of interpersonal and structural violence that contributes to gender and social inequalities and possibly detracts from health outcomes [26]. According to the authors, the term obstetric violence most directly highlights the necessity to evaluate biomedical systems and power structures within health facilities that might place women in harm’s way [26].

### Challenges to the definition of mistreatment

Overall, despite the increased discussion on terminology, there remain central challenges to defining mistreatment. First, the mistreatment of women during childbirth is both a form of gender-based violence and a form of institutional violence; mistreatment reflects gender inequalities not only within intra-hospital dynamics, but also in the allocation of health system resources and in societies more broadly. As such, mistreatment is fundamentally an interdisciplinary topic that requires attention from professionals in the fields of public health, human rights, medicine and medical ethics, gender studies, and other social sciences, such as anthropology, among others. Existing definitions based on literature reviews, such as the mistreatment typology developed by Bohren et al. [3], have largely focused on health research and may have overlooked legal documents or publications from other fields [26]. There is a need for interdisciplinary collaboration that can consolidate research and terminology from different fields of study to create a definition that is truly comprehensive.

Another challenge is establishing criteria for different forms of mistreatment that allow for the comparison of mistreatment between study sites, while also capturing the nuances of mistreatment unique to each location and study population. Critics have noted that the Bowser and Hill definitions and Latin American obstetric violence laws do not sufficiently specify what acts constitute abusive care [3, 27]. Without specific typology included in the category definitions, researchers using the disrespect and abuse categories have used different criteria and study methodologies to determine what constitutes physical abuse or non-dignified care, for example, and thus the comparability of studies has been limited [3]. However, as Jewkes and Penn-Kekana argue, the WHO’s mistreatment typologies might be too broad to be effective [1] and their framework might overlook the unique factors contributing to mistreatment against different populations, such as indigenous and Afrodescendant women in Latin America and the Caribbean.

Other overlooked nuances include the perspectives of mistreatment and norms among medical personnel and women using maternal health care services. Various research studies throughout the world have documented cases in which different forms of mistreatment become normalized to the extent that women or health providers do not view those issues as abusive [15, 28, 29]. However, to our knowledge, Freedman and colleagues have proposed the only framework that accounts for deviations between researchers’ definitions of mistreatment and women and medical personnel’s perspectives of those issues [4, 30]. These deviations carry important implications for the development of interventions to mitigate mistreatment and warrant consideration in the construction of mistreatment terminology.

Finally, researchers have struggled to create terminology that conveys mistreatment as a form of violence without assigning blame to health care providers as a group. For example, in their 2015 review on obstetric violence in Brazil, Diniz et al. highlighted that initial movements downplayed violence-centered terminology and focused on terms such as “humanizing childbirth” and “respectful birth,” in order to avoid hostility from health care providers [31]. Jewkes and Penn-Kekana also noted that the concept of intentional abuse of women during childbirth has received resistance from medical professionals, particularly those who already feel alienated working in low-resource health systems [1]. Nevertheless, despite this resistance, it is important to define and measure intentional violence as a component of mistreatment in order to increase accountability within health systems and accomplish meaningful change.

In addition to addressing the challenges listed previously, in this article we offer a few recommendations for the future development of mistreatment definitions and typologies. First, we recommend that future discussions consider elements of definitions where there is already consensus among researchers. Bowser and Hill’s disrespect and abuse categories, the WHO researchers’ mistreatment typologies, and Latin American obstetric violence frameworks share numerous common elements including parallels with violence against women, the medicalization of natural birth processes, multi-level sources of mistreatment, roots in gender inequalities, the potential for harm, and the threat to women’s rights and bodily integrity [13]. Second, we recommend that future discussions connect terminology with clear implications for action. For example, Freedman et al. also link each component of their disrespect and abuse framework with implications for formative research, epidemiological studies, and policy initiatives [4]. We believe that this approach would be beneficial for other frameworks and would aid the synthesis not only of terminology but also of measurement methodology.

## Measurement of mistreatment

This section includes a total of 46 research studies and 2 research protocols published between 2002 and 2017 that purposively seek to measure some aspect of mistreatment in clinical maternal health care settings. We subdivided the 48 publications into those that used quantitative (14 studies), qualitative (23 studies), and mixed methods (11 research studies and protocols). While the majority of these articles—38 articles—were published in English, another 7 were published in Portuguese, and 4 were published in Spanish.

Publications included a variety of countries throughout the world. Twelve studies sought to measure mistreatment within Latin America and the Caribbean, seven of which were qualitative and conducted in Brazil [32–38], one qualitative and one quantitative in Mexico [15, 39], two quantitative in Venezuela [40, 41], and one qualitative in Argentina [42]. Twenty-five publications focus on mistreatment in health facilities in one or more African countries, including Tanzania [28, 43–49], Nigeria [50–53], Ghana [54–57], Kenya [46, 58–60], Ethiopia [46, 61, 62], Tunisia [63], Guinea [64, 65], Mali [66], Madagascar [46], Rwanda [46], and South Africa [67]. Additionally, the two research protocols included in this review examine mistreatment in countries from both Africa and Asia, such as Kenya and Bangladesh [68], as well as Ghana, Guinea, Nigeria, and Myanmar [7]. Within Europe, countries of focus include Spain [69], Serbia and Macedonia [70], the United Kingdom [71], and Belgium, Iceland, Denmark, Estonia, Norway, and Sweden [72]. Other countries of focus include India [73], the United States [71, 74], Australia [71], and New Zealand [71].

### Quantitative methodologies for the measurement of mistreatment

Among the 14 quantitative study publications, summarized in Tables 1, 11 measure mistreatment exclusively through the collection and analysis of surveys and structured questionnaires [39–41, 43, 44, 52–54, 61, 72, 74]. While most of those cross-sectional studies rely on original instruments, two of the studies analyze survey data from larger cohort studies that examined women’s experiences of maternal health care—the Oregon Pregnancy Risk Assessment Monitoring System (PRAMS) study [74] and the Belgium, Iceland, Denmark, Estonia, Norway, and Sweden (BIDENS) cohort study group in six European countries [72]. In addition to those 11 studies, another two publications report data from an investigation in Kenya that used both surveys and structured direct observation checklists to assess mistreatment before and after an intervention to improve respectful maternity care [58, 59]. The remaining publication sought to report the prevalence of mistreatment in maternity facilities in five African countries by conducting structured clinical observations of 2164 labor and birth processes [46].

**Table 1:** Publications with Quantitative Methodologies for the Measurement of Mistreatment of Women during Childbirth

Author/Year/Location	Study Purpose	Study Population	Methodology	Detailed Methodology
Reis et al., 2005, Nigeria [53]	To conduct a population-based assessment of health workers’ attitudes and practices of discrimination against people with HIV in clinical settings.	1021 Nigerian health-care professionals (including 324 physicians, 541 nurses, and 133 midwives identified by profession) in 111 health-care facilities in four Nigerian states.	Cross-sectional study with two surveys	The first included 104 items with questions on respondent demographics; practices regarding informed consent, testing, and disclosure; treatment and care of patients with HIV/AIDS; and attitudes and beliefs about treatment and care of patients with HIV/AIDS including informed consent, testing, and disclosure. Treatment and care practices were assessed using Likert-type scales. Attitudes were assessed by a response of “agree” or “disagree” with statements regarding testing, treatment, and care of patients with HIV/AIDS. The second instrument had 103 items that asked about each facility’s capacity, resources, and policies from facility directors.
De Marco et al., 2008, Oregon, USA [74]	To assess women’s perceptions of being discriminated against during prenatal care, labor, and delivery; to assess the relationship between perceived experiences of discrimination and maternal/infant characteristics and frequency of infant checkup appointments.	5672 women living in Oregon who participated in 3 cohorts from the Pregnancy Risk Assessment Monitoring System (PRAMS) study.	Analysis of survey data from cohort study	The study analyzed data from the 1998–1999, 2000, and 2001 Oregon PRAMS study, which collected information about the attitudes and experiences of women through the continuum of maternal health care. To assess perceived discrimination in health care, the PRAMS asked women if they felt they had ever been treated differently by health care providers during prenatal care, labor, or delivery because of their race, culture, ability to speak or understand English, age, insurance status, neighborhood in which they lived, religious beliefs, sexual orientation or lifestyle, marital status, or desire to have an out-of-hospital birth. Response categories included “yes” and “no.”
Faneite et al., 2012, Venezuela [41]	To understand the extent to which health care providers understand national laws on obstetric violence and the mechanisms for reporting it.	500 health workers in three maternity hospitals in different parts of the country recruited through purposive sampling.	Cross-sectional study with survey	The study was conducted shortly after Venezuela passed legislation defining and outlawing obstetric violence. Participants completed the self-administered questionnaires in the hospitals where they worked. Questions consisted of yes/no questions about different types of obstetric violence and then open questions about the identification of who is responsible for violence and how to report it. Researchers were present while participants completed the survey. The study found little knowledge about details of the law or obstetric violence.
Terán et al., 2013, Venezuela [40]	To evaluate the perceptions of women using maternal health care services regarding experiences of obstetric violence.	425 postpartum women still admitted at one national hospital.	Cross-sectional study with survey	The survey included questions about women’s experiences of acts that would constitute obstetric violence according to the Venezuela Organic Law about the Rights of Women to a Life Free of Violence. Researchers analyzed the absolute frequency, percentages, and standard deviations of different experiences of obstetric violence.
Kruk et al., 2014, Tanzania [43]	To assess the frequency of reported disrespect and abuse during childbirth in rural areas of Tanzania.	1779 who had recent given birth at any of two district hospitals, five government health centers, and one government health dispensary and were recruited upon exiting the hospital.	Cross-sectional study with exit survey and follow-up survey	Questionnaires were administered to 1779 women upon exiting the health facilities, and follow-up surveys were administered to a random subset (593 women) of those initial participants between 5 and 10 weeks after giving birth. Surveys included 14 questions about potential experiences of abuses that were modeled on the categories of disrespect and abuse during childbirth developed by Bowser and Hill [5]. Responses were categorized as “experienced” or “not experienced.” Researchers determined frequencies and logistic regression to analyze associations between abusive treatment and individual and birth experience characteristics.
Abuya et al., 2015, Kenya [58, 59]	To assess levels of disrespect and abuse during childbirth as part of a pre and post intervention.	Women ages 15–45 who had delivered with 24–48 h at one of the 13 hospitals included in the intervention, regardless of their pregnancy outcome.	Cross-sectional study with exit survey, and structured observation checklists	Women were administered a questionnaire concerning mistreatment in general as well as typologies based on the categories developed by Bowser and Hill. General questions about mistreatment were constructed using a Likert scale, and questions about specific types of mistreatment were asked in a “yes” or “no” format. Midwife or nurse researchers also conducted structured observations of patient-provider interactions during labor using a checklist of seven categories of mistreatment. Observations measured both process (how patients are treated) and content (what they were told, revealing technical competency, accuracy of information and provision of essential information) of services.
Asefa and Bekele, 2015, Ethiopia [61]	To quantitatively determine the level and types of disrespect and abuse faced by women during childbirth at four health centers in Ethiopia.	173 who had recently had a vaginal delivery at one of the study sites were recruited via purposive sampling before being discharged from the health centers.	Cross-sectional study with exit survey	This cross-sectional study administered exit surveys that asked participants about experiences of 23 different types of mistreatment, which were grouped into seven categories. The types of mistreatment were determined through the seven categories of mistreatment defined by Bowser and Hill, and the verification criteria were developed as part of the Maternal and Child Health Integrated Program (MCHIP). Participants’ objective and subjective experiences of mistreatment were taken into consideration.
Kujawski et al., 2015, Tanzania [44]	To assess the association between perceived experiences of mistreatment and delivery satisfaction, perceived quality of care, and intention to deliver at the same facility in the future.	1388 postpartum women upon discharge from two hospitals in Tanzania. A subset of women received another survey 5–10 after the initial survey.	Cross-sectional study with survey	This study drew from a subset of participants in the Kruk et al. study in Tanzania [43]. Multivariable logistic regression models were used to assess the association between mistreatment and (1) satisfaction with delivery, (2) perceived quality of care for delivery, and (3) intent to use the same facility for a future delivery, controlling for confounders. Participants were asked to rate their satisfaction with delivery from four response choices: very satisfied, somewhat satisfied, somewhat dissatisfied and very dissatisfied. Women rated the quality of care they received for their delivery as excellent, very good, good, fair, or poor. Responses were dichotomized into excellent and very good compared to good, fair, and poor – categories based on past research, which indicated potential for courtesy bias among the population. The instrument drew from Bowser and Hill [5] and had “yes” and “no” questions about experiences of various forms of mistreatment.
Lukasse et al., 2015, Belgium, Iceland, Denmark, Estonia, Norway & Sweden [72]	To assess the impact of reported experiences of Abuse in Health Care (AHC) on women’s fear of childbirth and desire for a cesarean delivery.	6923 pregnant women attending routine prenatal care who participated in the Belgium, Iceland, Denmark, Estonia, Norway, and Sweden (BIDENS) cohort study.	Analysis of survey data from multi-country cohort study	The BIDENS study questionnaire included general questions on participants’ demographic and socioeconomic characteristics, mental health, and obstetric history. The questions on abuse were taken from the Norvold Abuse Questionnaire (NorAQ) and included three descriptive questions about experiences of AHC and one scaled-question about frequency of past experiences of AHC. Fear of childbirth (FOC) was assessed by the Wijma Delivery Expectancy/Experience Questionnaire (W-DEQ) version A. Women were also asked how they would prefer to give birth. Current and past experiences of AHC were associated with the other variables of interested.
Okafor et al., 2015, Nigeria [52]	To determine the prevalence of disrespect and abuse of women during childbirth at a large teaching hospital in Nigeria.	Convenience sample of 437 women who were accessing immunizations for their newborns within six weeks after delivery.	Cross-sectional study with survey	This cross-sectional study included a structured questionnaire with questions concerning disrespect and abuse. Mistreatment was grouped into seven categories based on the work of Bowser and Hill [5]. Participants were given yes/no as possible responses.
Rosen et al., 2015, Ethiopia, Kenya, Madagascar, Rwanda, Tanzania [46]	To gather the prevalence of mistreatment during childbirth at a diverse array of health centers in five countries in Africa.	Direct observations of 2164 labor and delivery processes.	Structured clinical observation checklists	Observations were part of a larger cross-sectional study called the Maternal and Child Health Integrated Program (MCHIP), conducted between 2009 to 2012. The observation checklist included 10 actions the providers should perform to guarantee that the client received respectful care. Elements of respectful care derived from the rights discussed in the *Respectful Maternity Care Charter*. For each country, percentage of women receiving these practices and delivery room privacy conditions were calculated. Observers’ open-ended comments were also analyzed to identify examples of mistreatment. Researchers tried to minimize the Hawthorne effect by letting health care providers know that the observations were anonymous and would not be reported if they did anything wrong. Obstetric professionals were trained as observers, used paper checklists or smartphones.
Valdez-Santiago, 2015, Mexico [39]	To characterize the types of abuse that occur in obstetric facilities in three hospitals of Morelos, Mexico.	512 women recruited immediately after giving birth in the postpartum areas of the study sites.	Cross-sectional study with survey	The study consisted of a structured questionnaire about experiences of abuse during labor, delivery, or postpartum care from the perspective of women. Questionnaire asked mostly yes/no questions.
Moyer et al., 2016, Ghana [54]	To examine what midwifery students learn and witness concerning respectful labor and delivery care.	853 students in the final year of their studies at 15 midwifery schools.	Cross-sectional study with computer-based survey	The cross-sectional study consisted of a computer-based self-administered survey. In addition to questions about demographics, the survey included questions about working in underserved areas, observations of respectful and disrespectful care during training, and perceptions of working conditions in the clinical settings where students train. For questions about witness events, participants had three choices of responses: “rarely or never,” “sometimes,” or “most of the time.” Surveys took between 30 and 45 min to complete, and participants received small monetary incentives.

The 14 quantitative studies use instruments that draw from a variety of conceptual frameworks. Primarily, six publications report study instruments based on Bowser and Hill’s categories of mistreatment during childbirth in health facilities [43, 44, 52, 58, 59, 61]. Another two investigations in Venezuela examine obstetric violence using the country’s legal definition as a framework for survey questions [40, 41]. The five-country, direct observation study drew from the *Respectful Maternity Care Charter* to create categories for direct observation checklists [46] and the six-country cohort study in Europe incorporated questions from the Norvold Abuse Questionnaire (NorAQ) [72]. To ask participants about different elements of mistreatment within their respective frameworks, surveys discussed in five publications incorporated Likert scale questions [44, 53, 54, 58, 59] and others asked simple “yes” or “no” (or in one case, “experienced” or “not experienced”) about their topics of focus [39, 41, 43, 52, 58, 59, 74].

Study populations also varied between the 14 quantitative research publications. The majority of the quantitative surveys sought to assess mistreatment from the perspectives of women before or immediately after discharge from maternity care facilities [39, 40, 43, 44, 58, 59, 61]. One of these studies also included a follow up survey for a subset of participants that was conducted around six weeks after participants’ initial interview [43]. Similarly, three studies surveyed women within six weeks after delivery or at multiple times during and after pregnancy to assess their perceived experiences of mistreatment throughout the maternal health care continuum [52, 72, 74]. In addition to the three publications describing direct observations of patients and health providers during labor and delivery, two studies surveyed medical providers to measure attitudes and discriminatory practices against people with HIV [53] and to evaluate participants’ understanding of national obstetric violence laws [41]. Finally, one study in Ghana surveyed midwifery students to assess gained insight on their exposure to and perceptions of mistreatment during training [54].

The 14 publications documented numerous strengths and limitations to quantitative approaches to measuring mistreatment. A key practical strength, the use of surveys, particularly patient exit surveys, has been shown to be relatively low-cost and to allow researchers to gather data from larger samples of study participants [61]. Calculating frequencies of mistreatment or discrimination in larger study populations, researchers were able to demonstrate the magnitude of the issue and highlighted specific areas in which policymakers and health leaders might develop interventions [43, 52, 59, 74]. Similarly, surveys with a large sample of medical providers and students created compelling evidence for areas to improve medical education [41, 53, 54]. Rosen and colleagues’ approach of direct observation checklists in a large multi-country study population was particularly powerful in highlighting the magnitude and frequency of mistreatment [46]. As Abuya and colleagues demonstrate, assessing both investigator-observed and participant-reported frequencies of mistreatment is an effective strategy to monitor the effects of interventions to improve respectful maternity care [58, 59].

Nevertheless, these quantitative methodologies may be limited in their ability to assess the prevalence of mistreatment or the root causes of mistreatment—given the inherent limitation of cross-sectional studies to determine cause-and-effect relationships. For example, various surveys in this article examine the prevalence of women’s perceived and reported experiences of mistreatment. However, the accuracy of results may be limited in circumstances when women are reluctant to report mistreatment for fear of repercussions from health providers [40, 59] or social desirability bias [53] or in settings where mistreatment has become normalized and accepted among patients [43, 44]. Participant responses may also be subject to recall bias [52, 74] and may report various forms of mistreatment that in fact reflect the same event [43]. However, a 2013 study in Tanzania conducted semi-structured interviews with women suffering from obstetric fistula, and concluded that women were able to recall their birth experiences accurately for numerous years after those experiences took place [45]. Another 2014 publication from Tanzania found that reported rates of disrespect and abuse were significantly higher among women participants who received a follow-up interview five to ten weeks after completing an initial exit survey [43]. While strategies that utilize surveys as well as direct observation checklists may help to address these issues, a key limitation remains in the abilities of quantitative studies to capture the complexities of mistreatment and to explain why participants’ perceived experiences might not align with observed measures of mistreatment; they also offer limited insight on the structural and interpersonal drivers of mistreatment—factors that must be addressed to accomplish sustainable change in health facility cultures and providers’ practices. Furthermore, none of the publications in this review attribute suboptimal maternal or infant health outcomes to experiences of mistreatment [43, 44, 72].

### Qualitative methodologies for the measurement of mistreatment

In addition to the quantitative investigations, this review found a total of 23 publications that reported data from qualitative methodologies, summarized in Table 2. While eight of the 23 publications gathered data primarily through semi-structured, in-depth interviews [28, 32–35, 38, 63, 67], one study used unstructured interviews exclusively [71] and one publication reported the results of focus group discussions [56]. Over half of the publications—13 articles—described studies that incorporated a variety of qualitative methodologies. For example, eight publications described the results of studies combing focus group discussions and interviews [45, 50, 51, 55, 57, 64, 65, 70]. Researchers in another three investigations utilized both unstructured participant observations and unstructured or semi-structured interviews [15, 36, 69]. Finally, two publications described the results of full day “workshops” in which participants participated in group discussions, role play, and brainstorming, among other activities [37, 42].

**Table 2:** Publications with Qualitative Methodologies for the Measurement of Mistreatment of Women during Childbirth

Author/Year/Location	Study Purpose	Study Population	Methodology	Detailed Methodology
Castro & Erviti, 2003, Mexico [15]	To shed light on various sociological factors that contribute to abuse during facility-based childbirth.	200 women who reported abuses in medical facilities in Mexico, and another 64 observations of patient-provider interactions during labor and delivery.	Unstructured birth testimonies and qualitative direct observation	The study consisted of three phases. First, researchers compiled approximately 200 testimonies from women who reported having experienced various types of mistreatment over a five-year period. Many testimonies were collected from other studies that did not directly intend to measure mistreatment. Second, testimonies were assessed using qualitative methodology, looking for patterns that emerge from the testimonies. Third, researched conducted direct observation in labor and delivery rooms in the two largest public hospitals in Cuernavaca.
Beck, 2004, New Zealand, USA, Australia, and United Kingdom [71]	To gain insight on the factors that contribute to reported traumatic experiences during childbirth.	38 women who had given birth within the previous 5 weeks to 14 years and reported emotional trauma from their birth experiences.	Written birth narratives	Participants were recruited online through Trauma and Birth Stress (TABS), a charitable trust based in New Zealand that helps women who have experienced PTSD during their childbirth experiences. Over an 18-month period, participants were asked to write and submit descriptions of their traumatic birth experiences, which were analyzed using Colaizzi’s method of thematic data analysis. Overall study methodology included descriptive phenomenology.
Steele & Chiarotti, 2004, Argentina [42]	To understand experiences of mistreatment for women who access post-abortion care.	300 women who received post-abortion care at public hospitals in Rosario, recruited through women’s community organizations, health workers, and community contacts. 31 women also gave testimonies.	Focus group discussions as part of full day workshops, role play of care scenarios, unstructured testimonies	Researchers organized 13 workshops, in which women participated in discussions about mistreatment and discrimination while seeking post-abortion care and engaged in role-play scenarios of receiving care. Investigators reported that role-play was effective in helping women discuss personal, sensitive, and taboo topics. 31 women gave in-depth testimonies of their experiences. Those interviews were unstructured.
d’Ambruoso et al., 2005, Ghana [57]	To gather women’s perspectives of their interactions with maternal health care providers and assess the acceptability of maternal health services.	Opportunistic sample of 21 women who were participating in another study in Ghana and had accessed maternal health services in the prior five years.	Focus group discussions, in-depth interviews	This study was part of the SAFE initiative, which aimed to increase the knowledge base on skilled birth attendance in various developing countries. The topics of semi-structured interviews and focus group discussions drew from the SAFE study framework and included: (1) Place of delivery, (2) Satisfaction with services, (3) Recommendations of care, (4) Recommendations of services to other women. Investigators first started using focus groups discussions, but abandoned that research strategy in favor of in-depth interviews when it became clear that women were not comfortable sharing personal, emotional experiences in front of a group. Interviews were conducted at a location of the participant’s choosing. The methodology was based on the constructivist paradigm toward enquiry.
Marque et al., 2006, Brazil [35]	To understand nurses’ perspectives of the humanization of childbirth.	12 nurses in obstetric wards recruited from study hospitals with convenience sampling.	Semi-structured interviews	Semi-structured interviews were categorized into (1) the meaning of “humanization” of childbirth, (2) examples of “humanizing” practices, (3) examples of “dehumanizing” practices, (4) role of nurses in humanizing care. Participant interviews were transcribed and analyzed using thematic analysis.
Muñoz, 2008, Spain [69]	To understand the forms of abuse that occurs during facility-based childbirth.	10 women who had just given birth at a hospital.	In-depth interviews and qualitative direct observations	The study consisted of in-depth interviews and participant observations with 10 women who had recently given birth in hospitals. Themes that emerged from the interviews included hierarchal and asymmetric power structures with doctors in hospitals, in which patients are passive, obedient, and submissive to the physicians’ demands. The author used patients’ descriptions of mistreatment as a framework to discuss gender inequalities within society.
Santos & Shimo, 2008, Brazil [36]	To understand women’s knowledge and consent to episiotomies during childbirth.	16 women who received episiotomies during their births at a teaching hospital.	Semi-structured interviews and unstructured observations	Semi-structured interviews asked women about their knowledge of episiotomies, whether someone asked their permission to perform an episiotomy, and information given to them by medical personnel about episiotomies. Interviews took place three days after the woman’s delivery and were audio-recorded and transcribed. Observations were conducted throughout participants’ labor, delivery, and postpartum period at the hospital.
Kruger et al., 2010, South Africa [67]	To understand the psychological experiences of giving birth and working as a nurse in South African maternity wards.	93 low-income, Afrikaans-speaking women who gave birth in maternity wards and 8 of the 12 of the maternity ward nurses.	Semi-structured interviews	Researchers conducted semi-structured interviews with maternity ward nurses to ask about the psychological experiences of their work. They were never directly questioned about violence or abuse of patients, though that was the topic of interest. Women who had recently delivered with interviewed and asked about experiences in which they did not like nurses’ behaviors. Participants participated in four interviews (during pregnancy, a few days after giving birth, three months after giving birth and six months after giving birth) by the same interviewer. Interviews were analyzed using social constructionist grounded theory and coded. The study helped to shed light on some of the structural drivers of mistreatment as it demonstrated that nurses’ aggressive behaviors might stem from hierarchal power structures within hospitals.
de Aguiar & d’Oliveira, 2011, Brazil [32]	To understand the dynamic between power structures and institutional violence in maternity care from the perspective of women using public services.	21 women who had given birth within three months before the interview.	Semi-structured interviews	Semi-structured interviews were conducted at participants’ homes to allow for participants’ comfort. The number of interviews was determined by when investigators thought saturation was reached. The interview guide contained open-ended questions that asked about access, quality of care, and previous experiences with public maternity hospitals, and women’s perceptions and experiences of mistreatment. Results shed light on how obstetric violence occurs against patients and how patients learn to adapt strategies of acceptance or resistance against mistreatment. Results also indicate that many patients come to trivialize and expect mistreatment as part of the care process.
Janevic et al., 2011, Serbia and Macedonia [70]	To develop a conceptual framework showing how different levels of racism occur in maternal health settings and affect access to maternal health care among Romani women.	71 Romani women who had given birth within the past year recruited through purposive sampling, 8 gynecologists, 11 key informant interviews from NGOs & state institutions recruited through snowball sampling.	Community-based participatory research study with focus groups and semi-structured interviews	Based on community-based participatory research principles, the study included focus group discussions with Romani women with questions on health knowledge and beliefs during pregnancy, what women did after they found out they were pregnant, and experiences during prenatal care and delivery. The study also included semi-structured interviews with gynecologists and key informants. For gynecologists, interview questions focused on the provider practices, their daily challenges, and experiences with Romani patients. For key informants from NGOs, interviews focused on barriers to maternal care for Romani women. Experiences of racism in maternal health care were organized into a framework of three categories: institutional racism, personally-mediated racism, and internalized racism.
de Souza et al., 2011, Brazil [38]	To explore how health care providers perceive the humanization of childbirth.	17 professionals who work in four hospitals, two public, one affiliated with SUS, and one not affiliated with SUS.	Semi-structured interviews	The study used semi-structured interviews to examine perceptions of the humanization of childbirth care process as defined by national legislation. Thematic analysis revealed three principle categories: (1) the meaning of humanization; (2) practice of humanized care, and (3) difficulties in practicing humanized childbirth care. The study showed deficiencies in healthcare infrastructure and medical training that leave personnel not fully equipped to provide humanized care.
Aguiar et al., 2013, São Paolo, Brazil [33]	To understand the dynamic between institutional violence, gender, and power structures in public maternity hospitals.	21 women who had given birth at public hospitals within three months, 18 health care workers recruited through snowball sampling.	Semi-structured interviews	Interview questions focused on participants’ experiences and definition of violence. Interviews were conducted a location of participants’ choosing outside of the hospitals. Transcripts were analyzed using thematic coding and considered the social position of the participants (gender, age, income, race, etc.). The study found that health providers acknowledged violent practices but didn’t see them as violence but as necessary in a ‘difficult’ context and for the patients ‘own good.
Moyer et al., 2013, Ghana [55]	To examine community and health providers attitudes towards mistreatment during delivery incorporating a human rights perspective.	128 community members, including women with their newborn infants, and 13 health care providers recruited purposively at hospitals and health facilities.	Semi-structured interviews and focus group discussions	Community member participants were identified through community key informants in two randomly selected zones of study areas. Topics for interviews and focus group discussions were constructed using the *Respectful Maternity Care Charter* with an additional category concerning traditional birth practices. Both interviews and FGDs were analyzed using NVIVO thematic analysis.
Mselle et al., 2013, Tanzania [45]	To identify potential weaknesses in acceptable and quality care for women who suffer obstetric fistula.	16 women who suffered obstetric fistula, 5 nurse-midwives, six husbands of affected women, and six community members.	Semi-structured interviews and focus group discussions	Data collection was carried out over two years at a Comprehensive Community Based Rehabilitation Center. Semi-structured interviews were conducted with women about their experiences of care and with nurse-midwives from different levels about their experiences in delivering care. Sample size was determined by when saturation was reached. Focus groups were conducted with community members and described hypothetical situations about women facing challenges in obtaining quality birth care. Focus groups were also conducted with women’s husband about the challenges their wives faced. The study documented poor quality of care and gained insights into why health care personnel do not care well for their patients.
Andrade & de Melo Aggio, 2014, Brazil [34]	To gain exploratory data about obstetric violence from the perspectives of women who receive institutional care.	Four women who had given birth in health facilities.	Semi-structured interviews	Semi-structured interviews were conducted at women’s houses until saturation level was reached. The interview guide was developed using principles established in the National Law for the Humanization of Childbirth.
Da Silva et al., 2014, Brazil [37]	To understand obstetric nurses’ perspectives, attitudes, and experiences of obstetric violence.	Obstetric nurses working in public and private health facilities.	“Brainstorming” sessions as part of full day meeting	Investigators conducted a full-day meeting with obstetric nurses. The purpose of the meeting was to “brainstorm” different ways in which obstetric violence occurs in hospitals based on the experiences and perceptions of nurse participants. Participant conversations were audio-recorded, transcribed, and analyzed using thematic analysis. The results were categorized into (1) violent utterances of health professionals to patients, (2) unnecessary and/or iatrogenic experiences procedures performed by health professionals and (3) the institutional unpreparedness with unstructured environment.
McMahon et al., 2014, Tanzania [28]	To gather data on the perspectives of mistreatment during childbirth among women and their male partners in order to inform policy and advance research .	Women who had delivered within the past 14 months at a health facility, their male partners, community leaders and health workers from 16 villages across 4 districts.	In-depth interviews	Community leaders and community health workers were identified purposively from areas that were identified as lacking access to health care. Participant recruitment emphasized women who had normal deliveries. All participants participated in interviews in a location of their choice. Interviews included open questions about perceptions of care and care seeking, and later asked probing questions about experiences of disrespect and abuse. Grounded Theory was used in developing codes from interviews a literature review was conducted based on code results. Results were also compared to categories of mistreatment described by Bowser and Hill [5].
Bohren et al. 2016, 2017, Nigeria [50, 51]	To understand the extent to which mistreatment becomes normalized among health care providers and women accessing health care.	41 women who had given birth within the past year, women who had given birth within the past five years, 17 nurse/midwives, 17 doctors, and 9 heath care administrators.	In-depth interviews and focus group discussions	The study included focus group discussions with women who had given birth within the past five years and in-depth-interviews with women who had given birth within the past year and healthcare providers. Topics included questions about experiences and perceptions of, and perceived factors influencing mistreatment during childbirth. IDI and FGDs were also presented with four scenarios of mistreatment: physical restraint, slapping, verbal abuse, and refusing to help a woman. Thematic analysis of results was conducted based on categories identified in systematic review by Bohren et al. [3]. Combined approach of asking general questions with very specific examples of mistreatment allowed for comparison with other studies and locations.
Rominski et al., 2016, Ghana [56]	To examine perspectives of mistreatment during childbirth among midwifery students in Ghana.	Midwifery students in the final year of training at 15 midwifery schools in all of the country’s regions.	Focus group discussions	Investigators constructed the discussion guide using Bowser and Hill’s categories of mistreatment and participants general perceptions of respectful maternity care [5] and the author’s previous study [55]. Recruitment involved contacting students who had previously participated in a related computer survey. Discussions began with definitions of respectful care. FGDs were transcribed verbatim, coded, and analyzed. The study found that students often tried to justify disrespectful care.
Amroussia et al., 2017, Tunisia [63]	To examine single mothers’ experiences and perceptions of giving birth in medical facilities.	11 single mothers who had given birth a public healthcare facility.	Semi-structured interviews	The study used a semi-structured interview guide. Questions drew from authors’ personal experience and knowledge and focused on four main topics: single women’s experiences of mistreatment, perceptions of the attitudes of health care personnel, barriers to accessing care, and self-perceptions as single mothers. Data was analyzed using feminist intersectional approach.
Balde et al., 2017, Guinea [64, 65]	To examine women’s and health care providers’ perceptions and experiences of mistreatment.	Women of reproductive age who had given birth within the past year and within the past five years, health care providers, and health facility administrators in an urban area and a semi-urban area of the country.	Focus groups and in-depth interviews	Participants were given four scenarios of mistreatment during childbirth including: (1) providers slapping a woman, (2) verbal abuse, (3) providers refusing to help patients, and (4) providers forcing women to give birth on the floor. Participants were also asked questions about the story of childbirth, perceptions and experiences of childbirth, elements of mistreatment, and factors that might influence how women are treated. Researchers used thematic analysis to understand the acceptability of and attitudes towards those instances of mistreatment, as well as the different types of mistreatment that may occur. Typologies of mistreatment drew from the Bohren et al. review [3]. Results found various forms of mistreatment and that both providers and women may accept mistreatment in certain scenarios.

As with the quantitative research studies, several investigations constructed their conceptual frameworks from the *Respectful Maternity Care Charter* [55], Bowser and Hill’s seven categories of mistreatment [28, 56], and legal frameworks on humanized maternity care [34, 35, 38]. Dissimilarly, four recent publications from the WHO multi-country tool assessment describe studies that construct and analyze questions using Bohren and colleagues’ mistreatment typologies [50, 51, 64, 65]. Researchers of the remaining qualitative publications constructed original frameworks based on literature reviews, personal experience, or results from previous studies regarding women’s perceptions of maternal health care.

Similar to the quantitative research publications included in this review, the qualitative investigations included a diverse range of study populations. Eight of the publications focused exclusively on the results of interviews or focus groups with women who gave birth in a medical facility, though the timing of data collection in these studies varied significantly. While three studies interviewed women immediately after childbirth [15, 34, 36], one study focused on women who had received post-abortion care [42], and another four studies collected data up to several years after women had given birth [32, 57, 63, 71]. Four other publications examined study populations that exclusively included health care personnel [35, 37, 38] or midwifery students [56]. Additionally, 10 publications included a mix of study populations, such as maternal health care providers and women who had recently given birth in a medical facility [33, 67]. The four studies using Bohren and colleagues’ mistreatment typology included health care administrators, medical providers, and women who had given birth within the previous five years [50, 51, 64, 65]. Similarly, four studies included medical providers, community members or key informants, and women who had recently given birth [55, 70], with two of those publications also gathering data from women’s husbands [28, 45].

The 23 publications included in this review evidenced numerous strengths and limitations to using qualitative methodology to assess mistreatment. A primary strength of interviews, participant observation, and focus groups is the ability to gain insight on the roots of mistreatment and nuances of mistreatment in different settings. By gathering participant perceptions and lived experiences of mistreatment, the qualitative studies produce information about power structures within health facilities and structural drivers of mistreatment [56, 67] and also illuminate possible mechanisms through which mistreatment might become normalized in health care settings [15, 28, 32, 33]. In-depth qualitative data on perceptions and experiences of mistreatment also provide insight on the complex intersections of inequalities that drive mistreatment and increase some populations’ vulnerability to discriminatory or abusive care [63, 70]. All of these strengths are particularly notable among studies that include multiple study populations, such as women, medical providers, health facility administrators, and community members [33, 67, 70]. Additionally, from a practical perspective, one author noted that role play and group exercises helped women to share more openly about sensitive experiences [42]. Finally, although these qualitative methodologies are not intended to produce statistics or prevalence estimates for mistreatment, various studies carry implications for political and programmatic action and for transferring research findings to other settings. Not only do some studies pinpoint areas in which future research is needed, but the qualitative studies also identify areas in which initiatives might seek to improve medical training and health facility culture [35, 55, 56].

Despite these strengths, these publications describe notable limitations to the qualitative studies. Primarily, various studies in the review have small sample sizes [32, 34, 69] and the methodology does not allow for comparative analyses between distinct groups of women [45, 70]. Another notable limitation, as various authors describe, women’s perceptions of their birth experiences are subjective and might not provide an accurate representation of the frequency of mistreatment [28, 45, 57]. Moreover, among studies that interview women several years after their birth experiences, results may be subject to recall bias [28, 32, 57]. Although several researchers attempt to prevent recall bias by interviewing women immediately after childbirth, results from this approach may be subject to social desirability or courtesy bias, as women with healthy newborns may not feel entitled to verbally express complaints [50, 57]. From a practical standpoint, all of the qualitative methodologies described in the review require an increased dedication of time and resources. Another study was forced to abandon their original design of focus groups to utilize in-depth interviews, as women participants felt uncomfortable sharing their birth stories in group settings [57]. Finally, as with the quantitative research publications, the 23 qualitative publications did not examine adverse health outcomes that might stem from experiences of mistreatment.

### Mixed methodologies for the measurement of mistreatment

Finally, 11 publications reported on studies that used or proposed mixed methodologies to measure mistreatment, summarized in Table 3. Two of these 11 publications described studies that conducted focus group discussions and structured survey questionnaires among women who had given birth in local medical facilities [75, 76]. Similarly, another two investigations relied primarily on survey questionnaires and in-depth interviews to gather data [62, 66]. The remaining publications describe multi-faceted studies that incorporated four or more methodologies to assess mistreatment among their respective study populations. For example, direct observation of labor and delivery room proceedings, focus groups, surveys for medical personnel, and patient exit surveys were used by five investigations and protocols [49, 68], three of which also conducted in-depth interviews [7, 47, 48]. Of note, this combination of methodologies is described in the WHO protocol for standardizing a tool for assessing mistreatment [7]. Another study protocol includes a review of hospital service statistics, patient records, and inventory, in addition to conducting surveys, focus groups, interviews, and labor and delivery room observations [60]. Finally, the final multi-faceted investigation used observations, interviews, and surveys, but also assessed mistreatment using mystery patients, quality schedules, and a review of hospital records [73].

**Table 3:** Publications with Mixed Methodologies for the Measurement of Mistreatment of Women during Childbirth

Author/Year/Location	Study Purpose	Study Population	Methodology	Detailed Methodology
Moore et al., 2002, Kenya and Bangladesh [68]	To discuss the adaptation process and pretest results for Maternity Care Provider Caring Behavior (MCPCB) assessment tools to improve “caring behaviors” of maternal health care providers during labor and delivery.	24 observations of labor and delivery processes, 22 midwives, 13 women who had recently given birth, and another 17 staff midwives and their instructors.	Direct observation tools, self-assessment survey for providers, focus group discussions, patient exit survey	The complete set of tools included four instruments: (1) the Maternity Care Provider Caring Behaviors Observational Assessment Tools; (2) maternity health care providers self-assessment tool; (3) provider focus group discussion guide; and (4) patient exit interview guide. The observational tool was developed to address the gaps between perceived issues and observed behaviors of medical providers. The eight original categories of provider ‘caring’ behaviors are: (1) Attending to human needs, (2) Being accessible to patient (3) Attending to emotional needs, (4) Respecting human rights and dignity, (5) Informing, explaining, and instructing, (6) Involving family members, (6) Incorporating cultural context, and (8) Minimizing negative behaviors. These categories were developed from the results of a literature review. Two hospitals in Kenya (one rural, one urban) and four facilities in Bangladesh (one rural, one urban, one public, one private) were selected to be nationally representative.
Hulton et al., 2007, India [73]	To assess the quality of care in maternity facilities in urban slum areas with a focus on patient experiences of respectful care and clinical practices of care	650 women living near study site health facilities who had given birth between 6 weeks to 8 months previously, hospitals records for all women at 6 hospitals who gave birth between 1996 and 1999, 70 women being discharged at 3 hospitals, 14 staff at 3 facilities.	Quality schedule, exit surveys, review of hospital records, mystery clients, community survey, direct observation, interviews	The study setting was an urban slum area with high poverty but high rates of institutionalized deliveries. Researchers developed a quality of care framework with emphasis on Experience and Provision of care. Subcategories of Experience of Care include: Human and physical resources; Cognition; Respect, dignity, and equity; and Emotional support. Subcategories of Provision of Care included: Human and physical resources; Referral system; maternity information systems; Use of appropriate technologies; and International recognized good practice.
Warren et al., 2013, Kenya [60]	To detail the protocol for study assessing mistreatment against women in childbirth before and after interventions.	Six health facilities and a large maternity hospital, health providers and managers in those facilities, national level managers and policy makers, women in labor and postpartum women and community members in the areas of the six facilities.	Focus group discussions, in-depth interviews, observations, service statistics, exit surveys, reviewing patient records, facility inventory	The evaluations includes 3–5 focus group discussions with women who gave birth at health facilities and at home, their family members, and local health workers that focus on perceptions, attitudes, and experiences of care. Also, researchers will hold in-depth interviews with 25 senior health managers and assess health facility practices through interviews with medical personnel, patient records, structured facility inventory, service statistics, observations of delivery and labor, and exit interviews with women patients ages 15–45. For interviews with medical personnel, Likert scales are used and providers are given the option to self-administer part of the interview. Researchers follow up with some women for case narratives on mistreatment. Using Bowser and Hill’s categories of mistreatment [5], researchers create a Construct Map with measurable elements of mistreatment to assess in study components and interventions.
Sando et al., 2014, Tanzania [49]	To examine if women with HIV experienced increased levels of mistreatment during childbirth at an urban hospital in Tanzania.	2000 postpartum women with and without HIV, 68 health care providers, and 200 observations of labor and delivery.	Direct observations, in-depth interviews, exit surveys, provider surveys	Researchers conducted interviews with women 3–6 h after delivery to capture their experiences of disrespect and abuse and assess their overall perceptions of care. Categories of mistreatment mentioned included in interviews parallels categories documented by Bowser and Hill [49]. Researchers also conducted direct observations of client-provider interactions around the time of childbirth. To understand provider attitudes and opinions, researches administered a structured questionnaire and conducted in-depth interviews. Topics of the surveys and interviews included definitions and perceptions mistreatment, training and practices for managing patients with HIV, and comfort level with women with HIV.
Vogel et al., 2015, Ghana, Guinea, Myanmar, Nigeria [7]	To explain a study protocol for assessing mistreatment against women in childbirth in four countries.	Medical personnel in maternity centers, facility administrators, and women (15–49) using those facilities recruited through purposive sampling.	Two-phase WHO study with in-depth interviews, focus group discussions, systematic review, exit surveys, direct observation	In the first phase, researchers will conduct a mixed methods systematic review concerning mistreatment during childbirth, and they will conduct focus group discussions as well as in-depth interviews with medical personnel, health facility administrators, and women who have used maternal healthcare facilities. Focus groups will occur with women who have given birth within 5 years, and interviews with women who have given birth within 12 months. These activities will occur in two maternal health facilities in each country (1 rural/peri-urban, 1 urban). Data from the first phase will be used to construct instruments for the second phase, in which the investigators will conduct surveys with postpartum women and observations of delivery room procedures and interactions. Categories of mistreatment addressed in the study are based on typologies outlined by Bohren et al. [3].
Warren et al., 2015, Mali [66]	To explore auxiliary midwives’ perspectives of mistreatment during childbirth in rural Mali.	67 rural auxiliary midwives recruited from a continuing education session at the regional reference hospital.	Survey, semi-structured interviews	The study consisted of a survey with 53 participants, and semi-structured interviews with 33 participants. Study components focused on descriptive norms of mistreatment (“what most people actually do”), as opposed to practices that participants reported doing themselves. Surveys incorporated open ended and Likert scale questions about respectful care practices and mistreatment, with a few questions specific to stage of labor. Semi-structured interviews focused more on participants’ own practices. Analysis considers categories of mistreatment defined by Bowser and Hill [5] and Freedman and Kruk [4].
Ratcliffe et al., 2016, Tanzania [47, 48]	To report the effects of a set of interventions to reduce abusive care during childbirth and measure levels of mistreatment before and after the interventions.	Women using the intervention hospital, medical providers and administrators at the hospitals.	Exit surveys, direct observations, follow up interviews, provider surveys, and provider in-depth interviews	The intervention consisted of an antenatal education program for women and workshops for medical providers. Baseline assessment strategies included: postpartum interviews, direct observations of labor and delivery, follow up interviews with women, provider questionnaires, and provider in-depth interviews. Intervention monitoring included: observations, pre-and-post tests for workshops and education, and post-workshop action plan. Post-intervention evaluation included: direct observation of labor and delivery, follow up interviews, and provider in-depth interviews.
Sheferaw et al., 2016, Ethiopia [62]	To validate a scale that measures women’s perceptions of respectful care.	509 women seeking postnatal care for infants within seven weeks after childbirth at public hospitals in three towns.	Literature reviews, in-depth interviews, survey tool	Items were generated through in-depth interviews with women who give birth in health facilities and a literature review. Face validity and content validity were assessed through expert review. The draft scale included 37 items and two additional measures of global satisfaction items, measured on a five- point Likert scale. The final scale with 15 items was loaded on four components. “The extracted components were labeled as friendly care, abuse-free care, timely care, and discrimination-free care. The final scale correlated strongly with the global satisfaction measures, indicating criterion-related validity of the scale.”
Sudhinaraset et al., 2016, India [75, 76]	To gain a comprehensive understanding of women’s perspectives of mistreatment during childbirth in health facilities.	418 women with a child under the age of 5, whose most recent birth had occurred at one of the local hospitals.	Focus group discussions, surveys	Focus groups were based on an interview guide with questions about migration, social connections with villages of origin and in the study site, experiences of mistreatment, health knowledge, access to health services, and gender norms and beliefs. Surveys asked questions about 11 types of mistreatment: discrimination, verbal abuse, threatening to withhold treatment, patient abandonment, neglect, refusing choice position, restricting birth companions, requesting bribes, and unnecessary separation from the baby. Questions draw from Cultural Health Capital framework.

As with qualitative and quantitative publications, investigations and protocols’ conceptual frameworks drew from original designs [62, 68, 73], the Bohren et al. mistreatment typologies [7], and Bowser and Hill’s categories of disrespect and abuse [49, 60, 66]. Another two publications incorporated the Cultural Health Capital framework into their design and analysis [75, 76]. Although the 11 publications discussed a variety of research methods, three investigations sought to gain comprehensive information from a single study population such as women who had given birth in a medical facility [62, 75, 76] and midwives [66]. Three other investigations used different methodologies to gather data from medical personnel and women who had received maternal health care at various time points (immediately after childbirth and various years afterwards) [49, 68, 73]. In addition to those study populations, three studies included health facility administrators [7, 47, 48] and one research protocol included national level policymakers, health sector leaders, and community members to the aforementioned groups [60].

Considering the results of these investigations, mixed methodology approaches appear to be the most effective strategy for measuring mistreatment of women during childbirth at health facilities. By using direct observations as well as surveys and interviews, mixed methods studies have demonstrated the potential to identify and close gaps between observed and reported frequencies of mistreatment [49]. Such multi-faceted approaches also may be particularly powerful for comprehensively measuring mistreatment before and after interventions [47, 48]; not only can they measure changes in the frequency of mistreatment, but mixed methods can also assess changes in hospital culture, perceptions of mistreatment, and structural drivers of mistreatment. Another strength, mixed methods approaches have potential to attain comprehensive data on mistreatment among specific populations such as women with HIV and women in contexts of poverty [49, 73]. Finally, mixed methods approaches have the potential to obtain comprehensive insight on the frequency of mistreatment and multi-level factors that drive mistreatment, such as structural factors contributing to abusive care, medical education and practice, the perceptions and knowledge of medical personnel, facility administrators, and patients, and implications of mistreatment for women’s health practices. Each of these factors will require consideration and attention in order to achieve meaningful impact on mistreatment.

However, despite these strengths, the use of mixed methods studies requires financial and human resources that may be difficult to attain in low-resource settings. The high level of required resources may also restrict the sample size of investigations and thus limit studies’ generalizability. Furthermore, studies that incorporate only surveys and qualitative methods may still lack observed measures of mistreatment [66, 76], and none of these 11 studies achieved measures of the impact of mistreatment on health outcomes.

## Discussion

### Challenges to measuring mistreatment

There remain overarching challenges in the measurement of mistreatment common to various methodological approaches. Although numerous cross-sectional studies, interviews, and focus groups have centered on women’s reported experiences of mistreatment, we argue that women’s voices and experiences should be kept central to the development of all future research initiatives and interventions to assess and mitigate mistreatment. As Jewkes and Penn-Kekana state in their 2015 commentary, “the essential feature of violence against women is that it stems from structural gender inequality, i.e., women’s subordinate position in society as compared to men” [1]. To address these inequalities, participatory initiatives should strive to enable women to have an active voice in determining the research and programmatic strategies to promote respectful maternal health care. Nevertheless, a key challenge lies in prioritizing women’s perspectives and experiences, while simultaneously accounting for underreporting or inconsistent reporting that may occur in contexts where mistreatment has become normalized. Even if experiences of certain forms of mistreatment do not affect women’s perceptions of care, those issues still require attention as they can perpetuate unjust power structures within health facilities and possibly detract from optimal health outcomes. Furthermore, inconsistent reporting may reduce the comparability of results and the accuracy of prevalence estimates.

One strategy to address this issue is the inclusion of outside perspectives in the form of direct observation checklists. However, this methodology may also be limited by investigator subjectivity, particularly relating to patient-provider interactions—such as language and tone—that may be more subject to different interpretations based on age, gender, and personal experience, thereby reducing the comparability of results. In their five-country study, Rosen et al. extensively trained medical professionals to act as observers, but later noted that observers’ prior professional experiences could still impact the reliability of results [46].

### Methodological considerations for the study of mistreatment

In this review, we detected several topics that existing research initiatives have not yet explored. While a few studies have examined discrimination and mistreatment against single mothers [63] or women with HIV [49], none of the publications in this review give special consideration to adolescents, who may be particularly vulnerable to discriminatory care [5]. Of note, mistreatment against adolescents may be especially relevant in Latin America and the Caribbean, which currently has the highest proportion of births attributed to adolescents of any region in the world [8]. Similarly, more research is needed to examine mistreatment among indigenous and minority ethnicity women in Latin America and the Caribbean, as regional research has discovered numerous issues of discrimination that may affect these populations within clinical settings [2]. Many of the investigations described in this review focus on peri-urban settings. However, to gain a more comprehensive understanding of mistreatment, more research is needed to assess mistreatment in rural areas and in contexts with different percentages of institutional deliveries.

Most importantly, there is a pressing need for research initiatives to determine the impact of mistreatment on the health outcomes of women and their newborns. As social epidemiologist Nancy Krieger argues in a 2012 analytic essay, approaches to studying discrimination and health should incorporate an integrated social-ecological perspective that considers both individual and structural level mechanisms through which discrimination may affect health outcomes [77]. Discrimination and mistreatment may affect health not only through direct interactions between healthcare providers and patients, but also through systems in which patients are exposed to structural level discrimination and subsequently come to embody mistreatment as suboptimal health outcomes. Evidence concerning the direct health impact of mistreatment will be invaluable in demanding urgency for the development of programs and policies to eliminate mistreatment.

This review also sheds light on practical implications for the timing and location of interviews, which may be applicable to various methodological approaches. First, existing studies that focus on women health service users as a key study population have collected data from a variety of points in time relative to women’s childbirth experiences. While various survey-based investigations have administered questionnaires as women are discharged from health facilities, numerous qualitative investigations have entailed in-depth or semi-structured interviews with women months and even years after their experiences of accessing care. As discussed previously, both approaches present strengths and limitations. While exit interviews may help to reduce recall bias, women participating in surveys and interviews conducted in health facilities may be reluctant to complain or may fear that health care providers may learn of their responses. As of now, the ways in which time and women’s life circumstances impact reporting and perceptions of mistreatment remain unclear. To better understand that issue and obtain more comprehensive information, future investigations should strive to interact with women at more than one point in time. That type of longitudinal approach could also aid in gathering information about the associations of mistreatment with maternal and infant health outcomes.

### Limitations

This review has various limitations that should be noted. First, literature searches were conducted in English, Spanish, and Portuguese and, although that is a strength, we may have missed relevant publications in other languages. Second, the review was not systematic and did not include specific criteria to ensure the exclusive inclusion of high quality articles. Third, the review only included a few sources from grey literature, and we recommend that future studies incorporate more publications from international agencies or non-governmental organizations in their analyses. Finally, we did not gather the specific instruments used by each investigation in this review. As many instruments drew from similar conceptual frameworks, a review of instruments would be valuable to assess the extent to which existing study results are already comparable.

## Conclusions

With this article, we aim at generating information that will contribute to discussions on the development of effective methodologies to measure mistreatment of women during childbirth. We recommend that future investigations strive to accomplish three goals: (1) measure the participant-perceived and the investigator-observed frequencies of mistreatment in maternal health settings, (2) assess the macro and micro level factors that drive mistreatment, and (3) gauge the impact of mistreatment on the health outcomes of women and their newborns. Such complex and comprehensive investigations will likely require an intense dedication of time and resources; consequently, we also advocate for increased funding and resources dedicated to the study of mistreatment, particularly in Latin America and the Caribbean, where more data are needed.

### Recommendations moving forward

A gold standard approach should incorporate a variety of methodologies and gather insight from multiple perspectives, such as from investigators, health facility personnel, and women using maternal health services. Mixed methodologies, though often the most expensive and logistically intensive, will constitute the most powerful strategy to gain comprehensive information on the complex, dynamic issue of mistreatment. Furthermore, although the current WHO protocol uses focus group discussions and in-depth interviews solely to inform the design of quantitative methodologies, we recommend the inclusion of qualitative methods in all phases of investigations that assess mistreatment. As we have argued before, mistreatment stems not only from the personal prejudices and behaviors of medical personnel, but also from unequal power dynamics within the medical field and “fractured health systems” in which mistreatment becomes embedded in the delivery of care [2]. Consequently, efforts to measure mistreatment and assess the effectiveness of interventions should consider structural factors and changes within the culture of medical care, which is why we recommend the use of qualitative methods as the most effective strategy to evaluate those complex topics.

To counteract issues related to subjectivity, we also recommend that investigations complement data based on women’s perspectives with information from the viewpoints of investigators, health care providers, and other stakeholders. Data from these additional viewpoints is critical not only to contextualizing women’s experiences of mistreatment, but also to gaining a comprehensive understanding of health facility culture and the mechanisms through which mistreatment takes place. Unfortunately, it may be impossible to determine completely objective estimates of the prevalence of mistreatment, given the many possible interpretations of some forms of abuse. However, we argue that the most accurate estimates will result from a triangulation of data from women’s reported experiences of mistreatment, from the experience of health care providers, and from observations of mistreatment conducted by researchers.

## Abbreviations

BIDENS: Belgium, Iceland, Denmark, Estonia, Norway, and Sweden Study Group
NorAQ: Norvold Abuse Questionnaire
PRAMS: Oregon Pregnancy Risk Assessment Monitoring System
USAID: United States Agency for International Development
WHO: World Health Organization
